# Na^+^/Ca^2+^ exchanger isoform 1 takes part to the Ca^2+^-related prosurvival pathway of SOD1 in primary motor neurons exposed to beta-methylamino-l-alanine

**DOI:** 10.1186/s12964-021-00813-z

**Published:** 2022-01-12

**Authors:** Tiziana Petrozziello, Francesca Boscia, Valentina Tedeschi, Anna Pannaccione, Valeria de Rosa, Angela Corvino, Beatrice Severino, Lucio Annunziato, Agnese Secondo

**Affiliations:** 1 Division of Pharmacology, Department of Neuroscience, Reproductive and Odontostomatological Sciences, School of Medicine, “Federico II” University of Naples, Via S. Pansini 5, 80131 Naples, Italy; 2 Department of Pharmacy, School of Medicine, “Federico II” University of Naples, Via D. Montesano 49, 80131 Naples, Italy; 3 IRCCS SDN, Via E. Gianturco 113, 80143 Naples, Italy

**Keywords:** L-BMAA, NCX1, SOD1, Calcium signaling, Neuroprotection, ApoSOD1

## Abstract

**Background:**

The cycad neurotoxin beta-methylamino-l-alanine (L-BMAA), one of the environmental trigger factor for amyotrophic lateral sclerosis/Parkinson-dementia complex (ALS/PDC), may cause neurodegeneration by disrupting organellar Ca^2+^ homeostasis. Through the activation of Akt/ERK1/2 pathway, the Cu,Zn-superoxide dismutase (SOD1) and its non-metallated form, ApoSOD1, prevent endoplasmic reticulum (ER) stress-induced cell death in motor neurons exposed to L-BMAA. This occurs through the rapid increase of intracellular Ca^2+^ concentration ([Ca^2+^]_i_) in part flowing from the extracellular compartment and in part released from ER. However, the molecular components of this mechanism remain uncharacterized.

**Methods:**

By an integrated approach consisting on the use of siRNA strategy, Western blotting, confocal double- labeling immunofluorescence, patch-clamp electrophysiology, and Fura 2-/SBFI-single-cell imaging, we explored in rat motor neuron-enriched cultures the involvement of the plasma membrane proteins Na^+^/Ca^2+^ exchanger (NCX) and purinergic P_2_X_7_ receptor as well as that of the intracellular cADP-ribose (cADPR) pathway, in the neuroprotective mechanism of SOD1.

**Results:**

We showed that SOD1-induced [Ca^2+^]_i_ rise was prevented neither by A430879, a P_2_X_7_ receptor specific antagonist or 8-bromo-cADPR, a cell permeant antagonist of cADP-ribose, but only by the pan inhibitor of NCX, CB-DMB. The same occurred for the ApoSOD1. Confocal double labeling immunofluorescence showed a huge expression of plasmalemmal NCX1 and intracellular NCX3 isoforms. Furthermore, we identified NCX1 reverse mode as the main mechanism responsible for the neuroprotective ER Ca^2+^ refilling elicited by SOD1 and ApoSOD1 through which they promoted translocation of active Akt in the nuclei of a subset of primary motor neurons. Finally, the activation of NCX1 by the specific agonist CN-PYB2 protected motor neurons from L-BMAA-induced cell death, mimicking the effect of SOD1.

**Conclusion:**

Collectively, our data indicate that SOD1 and ApoSOD1 exert their neuroprotective effect by modulating ER Ca^2+^ content through the activation of NCX1 reverse mode and Akt nuclear translocation in a subset of primary motor neurons.

**Video Abstract**

**Supplementary Information:**

The online version contains supplementary material available at 10.1186/s12964-021-00813-z.

## Background

Calcium (Ca^2+^) imbalance is now considered one of the key elements of the neurodegenerative process occurring in amyotrophic lateral sclerosis (ALS), a fatal adult-onset disease characterized by progressive degeneration of both upper and lower motor neurons [1, 2]. Accordingly, during the disease progression, dysfunctional Ca^2+^ homeostasis may lead to misfolding of several proteins [3], thus facilitating their toxic aggregation. Importantly, organellar Ca^2+^ homeostasis, with particular respect to the endoplasmic reticulum (ER), is compromised in ALS preclinical models and is now considered a relevant pathogenic mechanism of the disease [4, 5]. About 20% of cases of familial form (fALS) and 2–7% of sporadic form of ALS (sALS) are caused by mutations in the gene encoding the cytosolic Cu,Zn-superoxide dismutase (SOD1). This makes *sod1* the second most frequently mutated gene after *C9orf72* in ALS Caucasian patients [6–8] (http://alsod.iop.kcl.ac.uk/). While mutated SOD1 accumulates as unfolded trimers causing motor neuron degeneration [9], dysfunctional secretion of native wild-type SOD1 may also favor the neurodegeneration in ALS [10]. In fact, a chronic intraspinal infusion of wild-type SOD1 significantly delays disease progression in transgenic animals carrying mutant human SOD1^G93A^ [10]. Furthermore, mutant SOD1 may induce ER stress by targeting several molecular components of ER-associated degradation (ERAD) machinery [11]. On the other hand, a rapid exposure to wild type SOD1 may protect motor neurons against ER stress induced by the beta-methylamino-l-alanine (L-BMAA) [12], one of the cycad toxins causing the Guamanian form of ALS [13]. Interestingly, the activation of Akt/ERK1/2 pathway via a transient [Ca^2+^] rise may underline the protective effect of SOD1 [12]. Mechanicistically, this neuroprotective effect is independent from the catalytic activity of the enzyme, since the non-metallated form ApoSOD1, lacking dismutase activity, may induce protection of motor neurons from L-BMAA toxicity likewise SOD1 [12]. Therefore, considering that the neuroprotection exerted by SOD1 and ApoSOD1 may pass through a rapid and transient [Ca^2+^]_i_ increase, in the present study we investigated, by a pharmacological and siRNA approach, the involvement of the Na^+^/Ca^2+^ exchanger isoforms (NCXs), the cyclic adenosine diphosphate-ribose (cADPR) receptor and the purinergic receptor P_2_X_7_, most of which are implicated in the pathogenesis of ALS. Furthermore, the correlation between this neuroprotective increase in [Ca^2+^]_i_ and Akt activation has been investigated.

## Methods

### Reagents

Media, sera, and antibiotics for cell cultures were purchased from Life Technologies (Milan, Italy). Mouse monoclonal anti-p-Akt (#4051) and rabbit polyclonal anti-GRP78 (#3183) were from Cell Signaling Technology Inc. (Danvers, MA, USA). Rabbit polyclonal antibody against Akt1/2/3 (#sc-8312) was from Santa Cruz Biotechnology, Inc. (Dallas, TX, USA). Rabbit polyclonal antibody against NCX1 (#π11-13) was from Swant (Bellinzona, Switzerland), rabbit polyclonal anti-NCX3 antibody was done by Dr. K. Philipson (University of California, Los Angeles, CA, USA). Mouse monoclonal anti-SOD (#S2147), mouse monoclonal anti-α-tubulin (#T5168) and rabbit polyclonal anti-MAP2 (#M3696) antibodies were from Sigma-Aldrich (Milan, Italy). ECL reagents and nitrocellulose membranes were from GE Healthcare (Milan, Italy). SOD1, retinoic acid, L-BMAA, thapsigargin, ATP, H_2_O_2_, 8-bromo-cADPR, MK801, CNQX, oligomycin, 2-deoxy-d-glucose, 2′,7′-dichlorofluorescein diacetate (DCF-DA), PD98059, LY294002, and all other reagents were from Sigma-Aldrich (Milan, Italy). A430879 was a kind gift from Prof. S. Bruzzone (Department of Experimental Medicine, University of Genova, Genova, Italy). The inactive mutant HA-Aktk179M (Akt D–) plasmids were donated by Prof. P. Formisano (“Federico II” University of Naples, Naples, Italy). Fura-2/AM and SBFI/AM were from Life Technologies (Milan, Italy).

### Rat primary motor neurons

Motor neuron-enriched cultures were obtained from the spinal cord of 12–14-day-old Wistar rat embryos and cultured as previously described [12, 14]. Cytosine β-d-arabinofuranoside hydrochloride (Ara-C, 10 μM) was added at 4 and 8 DIV (days in vitro) to prevent non-neuronal cell growth. Primary motor neurons were kept at 37 °C in a humidified 5% CO_2_ atmosphere and used after 10–12 DIV. All the procedures were performed according to the experimental protocols approved by the Ethical Committee of “Federico II” University of Naples, Naples, Italy, and according to the guidelines and regulations by Italian Ministry of Health (D.Lgs. March 4th, 2014 from Italian Ministry of Health and DIR 2010/63 from UE).

### Hybrid cell line

NSC-34 motor neurons were grown in Dulbecco’s Modified Eagles Medium (DMEM) containing 10% fetal bovine serum (FBS), 2 mM l-glutamine, 100 IU/ml penicillin, and 100 μg/ml streptomycin, and kept in a 5% CO_2_ and 95% air atmosphere at 37 °C. Before each experiment, NSC-34 cells were differentiated in 10 µM retinoic acid for 48 h, thus triggering a typical neuronal phenotype [15].

### SOD1 inactivation

SOD1 was incubated with 200 mM H_2_O_2_ in 25 mM sodium bicarbonate buffer (pH 7.5) for 2 h at room temperature (RT). At the end, the reaction was stopped by adding 1000 U/ml catalase for 30 min at 37 °C. Finally, SOD1 activity was measured by the SOD assay kit purchased from Sigma-Aldrich (Milan, Italy), as previously described [12].

### [Ca^2+^]_i_ and [Na^+^]_i_ measurements

[Ca^2+^]_i_ was measured by single cell computer-assisted video-imaging in NSC-34 motor neurons and in primary motor neurons, as previously reported [16]. Results are presented as cytosolic Ca^2+^ concentration calculated by the equation of Grynkiewicz et al. [17, 18]. NCX activity was evaluated as Ca^2+^ uptake through the reverse mode by using a Na^+^-deficient *N*-methyl-d-glucamine (NMDG) solution (Na^+^-free) containing (in mM): 5.5 KCl, 147 NMDG, 1.2 MgCl_2_, 1.5 CaCl_2_, 10 glucose, and 10 HEPES (pH 7.4). The irreversible and selective inhibitor of the sarco(endo)plasmic reticulum Ca^2+^-ATPase (SERCA) thapsigargin (Tg; 1 μM) was added 10 min before the beginning of the recordings, as previously described [16]. NCX activity was calculated as Δ% of peak/basal [Ca^2+^]_i_ values after perfusion with a Na^+^-free solution. [Na^+^]_i_ measurement was performed by loading motor neurons with 10 μM SBFI/AM incubated in the presence of 0.02% pluronic acid for 1 h at 37 °C [19].

### Patch-clamp electrophysiology

NCX currents (I_NCX_) in motor neurons were recorded by patch-clamp technique in whole-cell configuration using the commercially available amplifier Axopatch200B and Digidata1322A interface (Molecular Devices), as previously described [16, 20, 21]. I_NCX_ was recorded starting from a holding potential of − 60 mV up to a short-step depolarization at + 60 mV (60 ms). A descending voltage ramp from + 60 to − 120 mV was applied. I_NCX_ recorded in the descending portion of the ramp (from + 60 to − 120 mV) was used to plot the current–voltage (I–V) relation curve. The I_NCX_ magnitude was measured at the end of + 60 mV (reverse mode) and at the end of − 120 mV (forward mode), respectively. To isolate I_NCX_, the same cells were recorded first for total currents and then for currents in the presence of Ni^2+^ (5 mM), a selective blocker of I_NCX_. To obtain the isolated I_NCX_, the Ni^2+^-insensitive unspecific currents were subtracted from the total currents (I_NCX_ = I_T_ − I_NiResistant_) [16, 20, 21]. Motor neurons were perfused with external Ringer's solution containing the following (in mM): 126 NaCl, 1.2 NaHPO_4_, 2.4 KCl, 2.4 CaCl_2_, 1.2 MgCl_2_, 10 glucose, and 18 NaHCO_3_ (pH 7.4). Twenty millimolar tetraethylammonium (TEA), 50 nM tetradotoxin (TTX), and 10 μM nimodipine were added to Ringer's solution to abolish potassium, sodium, and calcium currents. The dialyzing pipette solution contained the following (in mM): 100 K-gluconate, 10 TEA, 20 NaCl, 1 Mg-ATP, 0.1 CaCl_2_, 2 MgCl_2_, 0.75 EGTA, and 10 HEPES (pH 7.2). Membrane capacitance was calculated according to the following equation: C_m_ = τ_c_·I_o_/ΔE_m_(1 − I_∞_/I_o_), where C_m_ is membrane capacitance, τ_c_ is the time constant of the membrane capacitance, I_o_ is the maximum capacitance current value, ΔE_m_ is the amplitude of the voltage step, and I_∞_ is the amplitude of the steady state current [19].

### Immunocytochemistry

Motor neurons were cultured on glass coverslips for 12 days. Then, cells were rinsed twice in cold 0.01 M PBS (pH 7.4) and fixed in 4% (w/v) paraformaldehyde (Sigma-Aldrich, Milan, Italy) for 20 min at RT. After three washes in PBS, cells were blocked with 3% (w/v) BSA and 0.05% Triton-X (Bio-Rad, Milan, Italy) for 1 h at RT. Coverslips were then incubated overnight at 4 °C with the following primary antibodies: rabbit polyclonal antibody against NCX1 (#π11-13, Swant, Bellinzona, Switzerland), rabbit polyclonal antibody against NCX3 (Dr. K. Philipson Laboratory, University of California, Los Angeles, CA, USA), mouse monoclonal antibody against SOD (#S2147, Sigma-Aldrich, Milan, Italy), mouse monoclonal antibody against p-Akt (#4051, Cell Signaling Technology Inc., Danvers, MA, USA), or rabbit polyclonal antibody against MAP2 (#M3696, Sigma-Aldrich, Milan, Italy). After three washes in PBS, coverslips were incubated in the dark with the corresponding secondary antibodies for 1 h at RT. Dapi was used to stain nuclei. Images were acquired by using a Zeiss LSM 700 laser (Carl Zeiss) scanning confocal microscope.

### Small interfering RNA

NCX1 and NCX3 knocking down was obtained by siRNA duplex against NCX1 or NCX3 and their non-targeting control (Qiagen, Milan, Italy), as previously described [22, 23]. MEK1 downregulation was achieved by using a specific siRNA against MEK1 and its non-targeting Control (Dharmacon, Lafayette, CO, USA), as previously reported [12]. Motor neurons were transfected for 5 h with each duplex at a final concentration of 10 nM using HiPerFect transfection reagent (Qiagen, Milan, Italy).

### L-BMAA treatment and cell viability measurement

Primary cultures of motor neurons were exposed to 300 μM L-BMAA for 48 h. SOD1 (400 ng/ml) or ApoSOD1 (400 ng/ml) were added in fresh medium 10 min before L-BMAA addition, while the specific NCX1 activator, CN-PYB2 (10 nM) [24], was added in fresh medium together with the neurotoxin. After 48 h exposure to L-BMAA, mitochondrial activity was evaluated by the MTT (3[4,5-dimethylthiazol-2-yl]-2,5-diphenyl-tetrazolium bromide) assay. Data are expressed as a percentage of cell survival of control cultures.

### Western blotting

After treatments, cells were lysed in ice-cold lysis buffer containing 20 mM Tris–HCl (pH 7.5), 10 mM NaF, 1 mM phenylmethylsulfonyl fluoride, 1% NONIDET P-40, 1 mM Na_3_VO_4_, 0.1% aprotinin, 0.7 mg/ml pepstatin and 1 µg/ml leupeptin. Protein concentration of each sample was determined by the Bradford method [25]. Proteins (50 µg) were separated on 10% SDS–polyacrylamide gels and transferred onto Hybond ECL nitrocellulose membranes (GE Healthcare, Milan, Italy). Membranes were blocked with 5% non-fat dry milk in 0.1% Tween 20 (Sigma-Aldrich, Milan, Italy) (2 mM Tris–HCl and 50 mM NaCl, pH 7.5) for 2 h at RT and then incubated overnight at 4 °C in the blocking buffer containing the mouse monoclonal antibody against p-Akt (1:1000) or the rabbit polyclonal antibody against GRP78 (1:1000). Membranes were then re-blotted with the rabbit polyclonal antibody against Akt1/2/3 (1:1000) or with the mouse monoclonal anti–α-tubulin (1:2000) antibody. Immunoreactive bands were detected with the ECL reagent (GE Healthcare, Milan, Italy) and then the optical density of the bands was determined by Chemi-Doc Imaging System (Bio-Rad, Milan, Italy).

### Chemical hypoxia

Chemical hypoxia was reproduced by adding to the NSC-34 cells 5 gμ/ml oligomycin (an oxidative phosphorylation inhibitor) plus 2-deoxy-d-glucose (a glycolysis inhibitor) in a glucose-free medium, containing (in mM): 145 NaCl, 5.5 KCl, 1.2 MgCl_2_, 1.5 CaCl_2_ and 10 HEPES (pH 7.4) for 45 min. Control cells were exposed to a Normal Krebs medium, containing (in mM): 145 NaCl, 5.5 KCl, 1.2 MgCl_2_, 1.5 CaCl_2_, 10 glucose, and 10 HEPES (pH 7.4) for 45 min, as previously described [16].

### Reactive oxygen species (ROS) production

2′,7′-dichlorofluorescein diacetate (DCF-DA) was used to detect ROS production in differentiated NSC-34 motor neurons. At the end of the experiments, DCF-DA fluorescence was acquired by a Nikon Eclipse 400 microscope (Nikon Instruments) equipped with a CCD digital camera (Coolsnap-Pro, Media Cybernetics).

### Statistical analysis

Data are expressed as mean ± S.E.M. Statistical comparisons between controls and treated experimental groups were performed using the one-way ANOVA, followed by Newman-Keuls test. P < 0.05 was considered statistically significant.

## Results

### Plasma membrane Na^+^/Ca^2+^ exchanger isoform 1 (NCX1) mediates a rapid [Ca^2+^]_i_ increase induced by SOD1 and ApoSOD1 in rat primary motor neurons

**Fig. 1:**
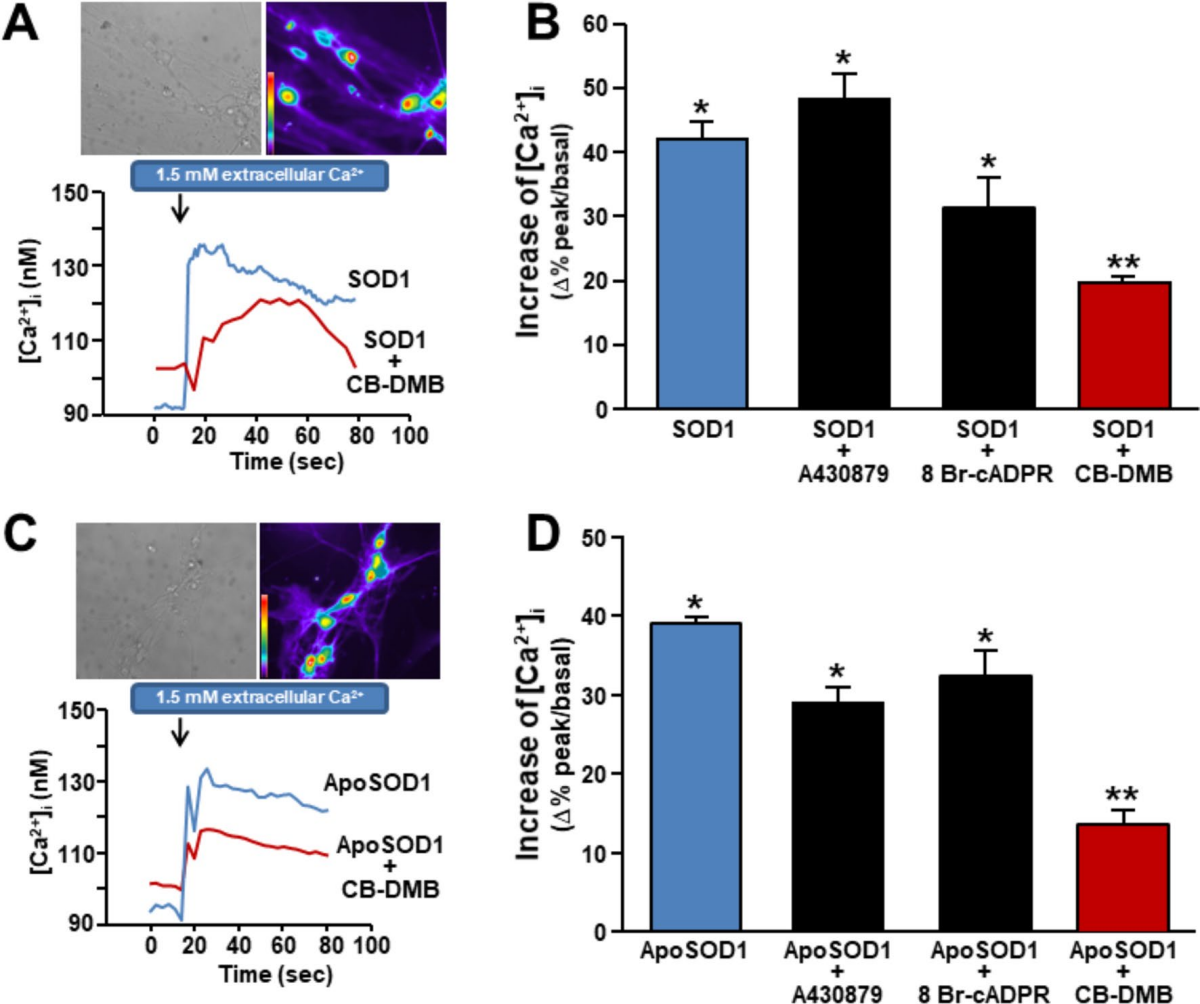
Effect of the pharmacological inhibitors of P_2_X_7_, cADP-ribose and NCX on SOD1- and ApoSOD1-induced [Ca^2+^]_i_ increase in rat primary motor neurons. **A** Superimposed representative traces of the effect on [Ca^2+^]_i_ of SOD1 alone (400 ng/ml) or in combination with CB-DMB (1 µM) in Fura-2-loaded primary motor neurons. **B** Quantification of the effect of SOD1 (400 ng/ml) alone (n = 30 cells) and in the presence of CB-DMB (1 µM) (n = 25 cells), the specific antagonist of P_2_X_7_ receptor, A430879 (1 μM) (n = 35 cells) or the cell permeant antagonist of cADP-ribose, 8-bromo-cADPR (10 μM) (n = 28 cells). Primary motor neurons were pre-incubated with 8-bromo-cADPR, A430879 or CB-DMB for 10 min before [Ca^2+^]_i_ recordings. All the experiments were repeated at least three times; *p < 0.05 versus internal control (basal values of [Ca^2+^]_i_), **p < 0.05 versus internal control and SOD1 alone. **C** Superimposed representative traces of the effect on [Ca^2+^]_i_ of ApoSOD1 alone (400 ng/ml) or in combination with CB-DMB (1 µM) in Fura-2-loaded primary motor neurons. **D** Quantification of the effect of ApoSOD1 (400 ng/ml) alone (n = 29 cells) and in the presence of CB-DMB (1 µM) (n = 30 cells), the specific antagonist of P_2_X_7_ receptor, A430879 (1 μM) (n = 35 cells) or the cell permeant antagonist of cADP-ribose, 8-bromo-cADPR (10 μM) (n = 30 cells). Primary motor neurons were pre-incubated with 8-bromo-cADPR, A430879 or CB-DMB for 10 min before [Ca^2+^]_i_ recordings. All the experiments were repeated at least three times on different cultures; *p < 0.001 versus internal control (basal values of [Ca^2+^]_i_), **p < 0.05 versus internal control and ApoSOD1 alone

In consideration of the modulatory role exerted on Ca^2+^ signaling by purinergic P_2_X_7_ receptor [26], cADP-ribose receptor [27, 28], and Na^+^/Ca^2+^ exchanger (NCX) [29–31], rat primary motor neurons were exposed to SOD1 or ApoSOD1 in presence of their specific antagonists [32–34] A430879 (1 μM), 8-bromo-cADPR (10 μM), or CB-DMB (1 μM). These pharmacological tools were used at the respective IC_50_ for the proposed targets. Our results indicated that only the NCX pan inhibitor CB-DMB significantly reduced the early increase of [Ca^2+^]_i_ induced by SOD1 (Fig. 1A, B) and ApoSOD1 (Fig. 1C, D). In contrast, A430879, blocking P_2_X_7,_ and the cell permeant 8-bromo-cADPR, that inhibits cADP-ribose action, did not modify SOD1- (Fig. 1B) and ApoSOD1-induced [Ca^2+^]_i_ rise (Fig. 1D). This may suggest the involvement of NCX in the upstream mechanism of SOD1 and ApoSOD1 and possibly highlights the participation of the exchanger in their prosurvival effects against L-BMAA toxicity.

### The activation of NCX1 reverse mode induced by SOD1 and ApoSOD1 is due to the [Na^+^]_i_ accumulation in rat primary motor neurons

**Fig. 2:**
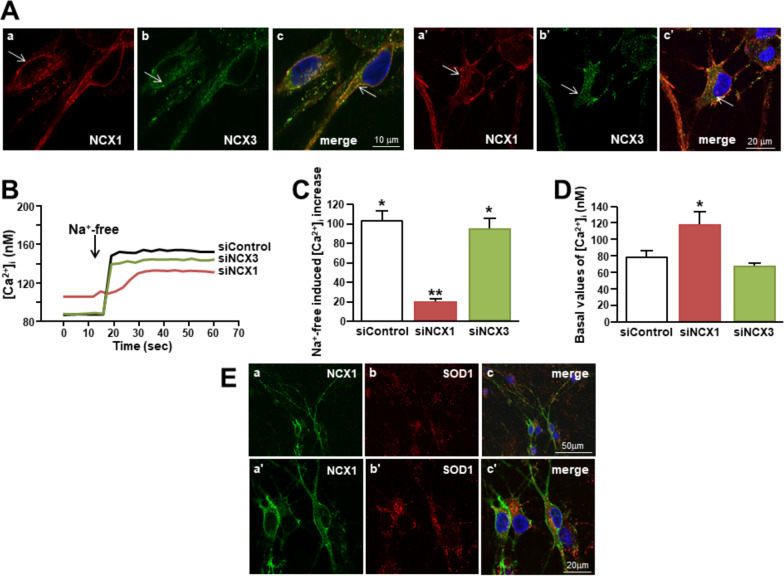
NCX1 and NCX3 expression and function in rat primary motor neurons. **A** Immunolocalization of NCX1 and NCX3 isoforms in two different motor neurons within the same culture. **B** Superimposed representative traces of the effect of Na^+^-free on [Ca^2+^]_i_ in motor neurons singly transfected with siControl, siNCX1 or siNCX3 (both at 10 nmol/L for 48 h). For details, please refer to Material and Methods. **C** Quantification of B expressed as Δ% of increase. All the experiments were repeated at least three times; *p < 0.001 versus internal control (basal values of [Ca^2+^]_i_), **p < 0.05 versus All. **D** Quantification of basal values of [Ca^2+^]_i_ of the treatments of B. *p < 0.05 versus All. **E** Immunolocalization of NCX1 and SOD1 in motor neuron-enriched culture at two different magnifications

In order to identify which isoform of NCX was involved in the Ca^2+^-dependent neuroprotective mechanism elicited by SOD1 and ApoSOD1, we analyzed the expression and activity of NCX1 and NCX3 isoforms in motor neuron-enriched cultures. As shown by confocal analysis in Fig. 2A, NCX1 and NCX3 isoforms were both significantly expressed in motor neurons. Of note, NCX1 was expressed in ~ 100% of MAP2-positive motor neurons of the enriched cultures, the 19% ± 2 of which expressed NCX1 at higher level (Additional File 1). Interestingly, NSC-34 cells significantly expressed the two exchanger isoforms with the same localization (Additional File 1). Of note, in these clonal motor neurons, SOD1 induced a significant increase in [Ca^2+^]_i_ that was prevented by the NCX inhibitor CB-DMB but not by AMPA and *N*-methyl-d-aspartate (NMDA) receptor inhibitors CNQX and MK801, respectively (Additional File 1).

**Fig. 3:**
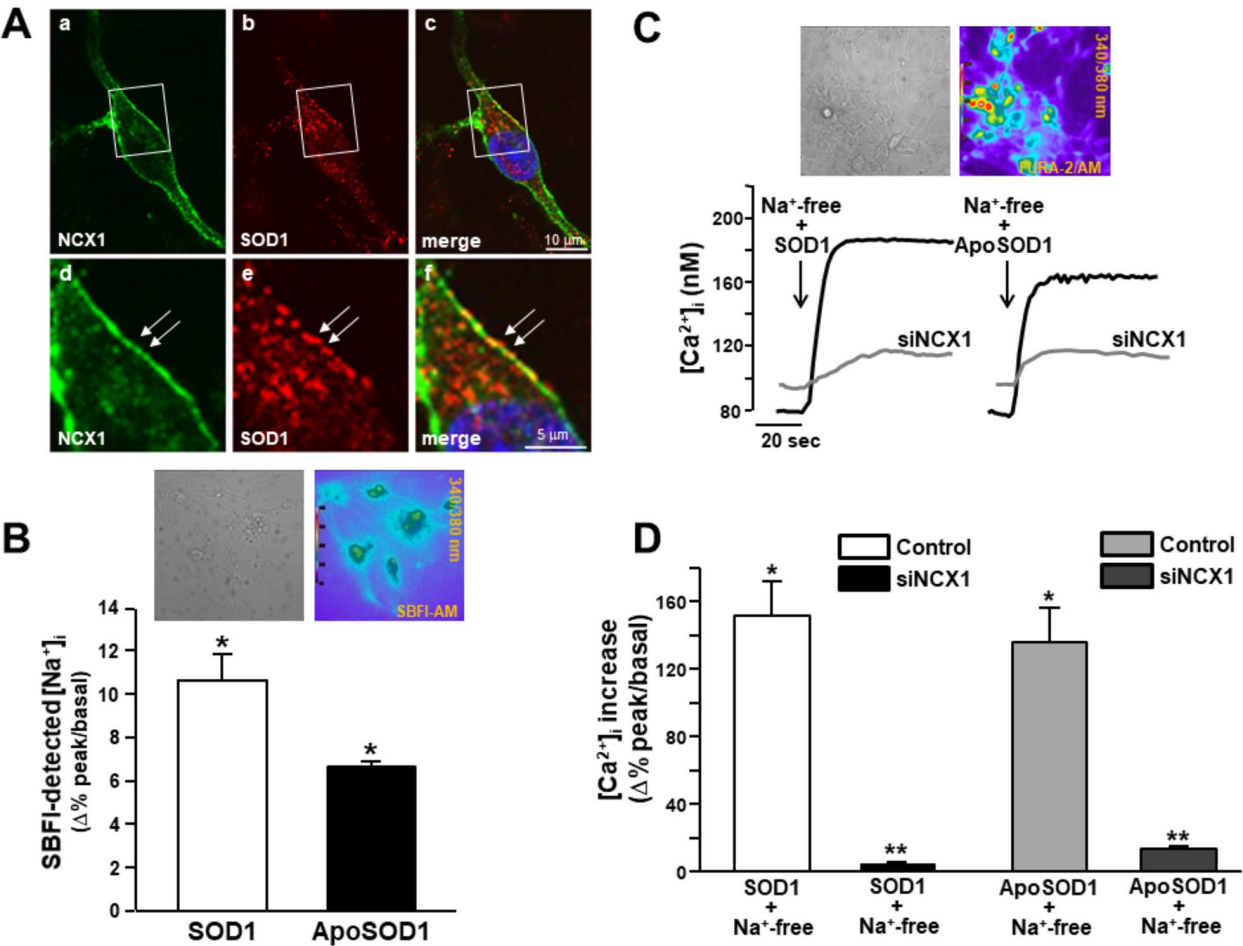
Effect of SOD1 and ApoSOD1 on NCX1-mediated [Ca^2+^]_i_ influx and [Na^+^]_i_ in rat primary motor neurons. **A** Co-localization between plasmalemmal NCX1 and SOD1 in a representative rat primary motor neuron at two different magnifications (see arrows). **B** Quantification of SOD1 and ApoSOD1-induced [Na^+^]_i_ increase in SBFI-loaded motor neurons (see representative images on the top). Data are quantified as Δ% of increase in 35 cells for each group. All experiments were repeated at least three times; *p < 0.05 versus internal control (basal values of [Na^+^]_i_). **C** Superimposed representative traces of the effect on [Ca^2+^]_i_ of SOD1 (400 ng/ml) in Na^+^-free solution and ApoSOD1 (400 ng/ml) in Na^+^-free solution both perfused on siControl neurons or siRNA-treated neurons against NCX1 (10 nmol/L for 48 h) loaded with Fura-2 (see representative images on the top). **D** Quantification of the effect of C reported as Δ% of increase in 40 cells for each group. All the experiments were repeated at least three times; *p < 0.001 versus SOD1 or ApoSOD1 alone (in the presence of external Na^+^); **p < 0.05 versus “SOD1 + Na^+^-free” or “ApoSOD1 + Na^+^-free”

However, in primary motor neurons and in differentiated NSC-34 cells, NCX1 was detected only on plasma membrane of the soma and neuronal processes, while NCX3 was mostly present in the whole intracellular compartment (Fig. 2; Additional File 1). In primary motor neurons, the intracellular localization of NCX3 was prevalent in some motor neurons resembling motor neurons 2. Therefore, a clear co-localization between the two isoforms was only marginally observed (Fig. 2A; Additional File 1). Then, NCX activity was studied by exposing Fura-2/AM-loaded motor neurons to a Na^+^-free solution forcing the exchanger to operate in the reverse mode-mediating [Ca^2+^]_i_ increase (Fig. 2B, C). However, NCX1 knocking down produced by siNCX1 completely abolished Na^+^-free-induced [Ca^2+^]_i_ rise, while NCX3 knocking down did not (Fig. 2B, C). Interestingly, in siNCX1-treated neurons a significant increase of basal [Ca^2+^]_i_ was detected if compared to control (i.e. siControl-treated neurons) (Fig. 2D). Moreover, SOD1 immunosignal was detected in NCX1-positive motor neurons (Fig. 2E) in which a significant co-localization between NCX1 and endogenous SOD1 was observed sometimes at plasma membrane level (see arrows of Fig. 3A). Moreover, in SBFI-loaded motor neurons, SOD1 (400 ng/mL) induced a significant increase in [Na^+^]_i_ when compared to untreated controls (Fig. 3B). The same [Na^+^]_i_ rise was detected after a brief exposure to ApoSOD1 (400 ng/mL) (Fig. 3B).

Furthermore, NCX activity was potentiated by both SOD1 and ApoSOD1 added to a Na^+^-free solution compared with control neurons exposed to Na^+^-free alone (Fig. 3C, D). However, this Na^+^-free-dependent activation of NCX was abolished in motor neurons previously silenced for NCX1 (Fig. 3C, D).

### NCX1 reverse mode induced by SOD1 and ApoSOD1 determines ER Ca^2+^ entry in rat primary motor neurons

**Fig. 4:**
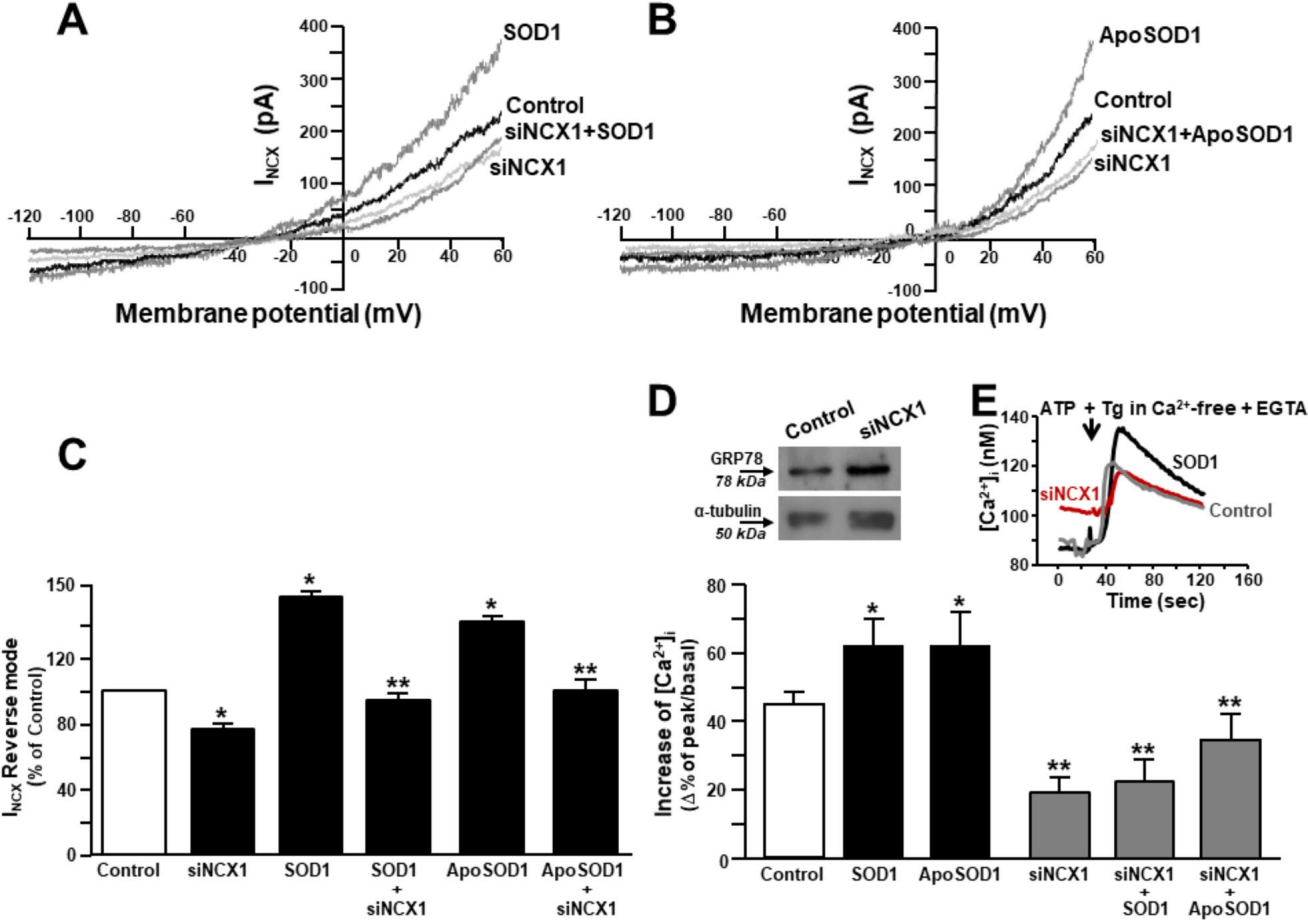
Effect of SOD1 and ApoSOD1 on NCX1-mediated currents (I_NCX_) and ER Ca^2+^ content in rat primary motor neurons. **A** Superimposed traces of I_NCX_ recorded by patch-clamp in rat primary motor neurons perfused with SOD1 and previously transfected with siControl (control) or siRNA against NCX1 (siNCX1; 10 nmol/L for 48 h). **B** Superimposed traces of I_NCX_ recorded by patch-clamp in rat primary motor neurons perfused with ApoSOD1 and previously transfected with siControl (control) or siRNA against NCX1 (siNCX1). **C** Quantification of A (n = 20 cells for each group) and B (n = 15 cells for each group). All the experiments were repeated at least three times; *p < 0.05 versus siControl (control); ** p < 0.05 versus SOD1 or ApoSOD1 alone. **D** Effect of SOD1 and ApoSOD1 preincubation (10 min) on ER Ca^2+^ content in the absence or presence of siNCX1 (10 nmol/L for 48 h). ER Ca^2+^ content has been measured in Fura 2-loaded motor neurons by adding ATP (100 µM) and thapsigargin (1 µM) in a Ca^2+^-free solution containing EGTA. Quantification has been reported as Δ% of increase in n = 30 cells for each group recorded in 3 independent experiments. *p < 0.05 versus siControl (control); **P < 0.05 versus All. **E** Superimposed representative traces of control motor neurons (siControl), motor neurons preincubated with SOD1 (10 min) or siNCX1-transfected neurons exposed to ATP and thapsigargin in a Ca^2+^-free solution containing EGTA. Inset: Representative Western blotting of GRP78 expression in control (siControl) and siNCX1-treated motor neurons (protein expression in control = 1 ± 0.001 and in siNCX1 = 3 ± 0.004 *p < 0.05)

To study the mechanism of action of SOD1, NCX current was recorded by patch-clamp electrophysiology in whole cell configuration (Fig. 4). SOD1, as well as its metal-free form ApoSOD1, determined a significant increase of NCX reverse mode measured at + 60 mV (Fig. 4A–C). On the other hand, NCX forward mode, measured at -120 mV, was unaffected by SOD1 or ApoSOD1 perfusion (Fig. 4A–C). Moreover, the knocking down of NCX1 by siNCX1 not only reduced NCX total current in motor neuron-enriched cultures but also counteracted the increase of NCX reverse mode induced by SOD1 (Fig. 4A, C) or ApoSOD1 (Fig. 4B, C). Of interest, the preincubation with SOD1 or ApoSOD1 enhanced ER Ca^2+^ content that was measured at cytosolic level as ER Ca^2+^ release after the perfusion with the sarco(endo)plasmic reticulum ATPase inhibitor thapsigargin (Fig. 4D, E). Interestingly, this SOD1-induced ER Ca^2+^ accumulation, as well as that produced by ApoSOD1, was prevented by siNCX1 (Fig. 4D). Of note, siNCX1 reduced ER Ca^2+^ content above the control level (Fig. 4D). Accordingly, in siNCX1-treated motor neurons the ER stress marker GRP78 was upregulated compared with siControl (*inset* Fig. 4D).

### Nuclear localization and phosphorylation of Akt induced by SOD1 and ApoSOD1 depend on NCX1 activation

**Fig. 5:**
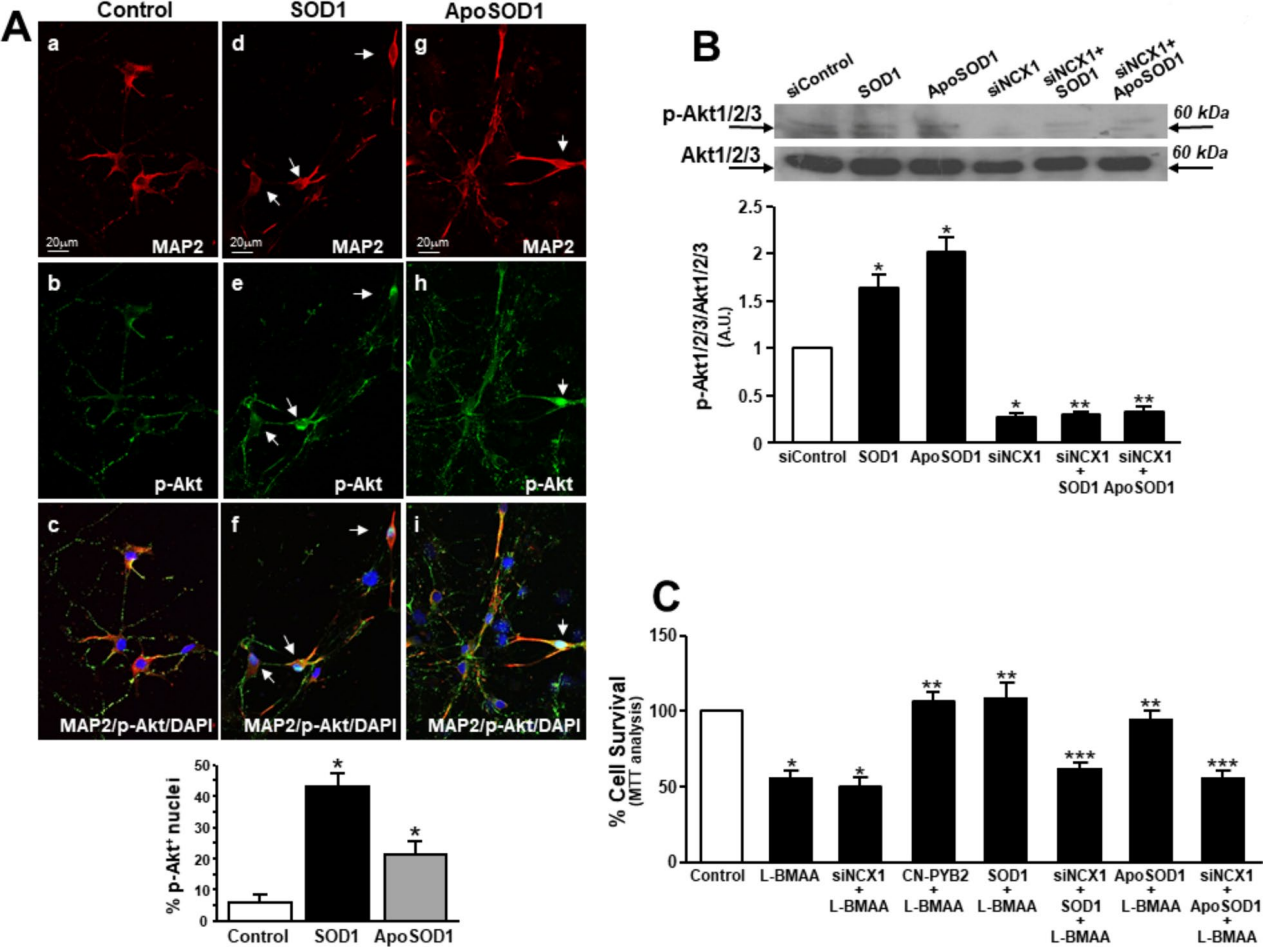
Effect of SOD1 and ApoSOD1 on phospho-Akt expression and localization in rat primary motor neurons. **A** Immunolocalization of phospho-Akt (p-Akt) in MAP2-positive cells within a motor-neuron enriched culture under control conditions (a–c), exposed to SOD1 (400 ng/ml/10 min) (d–f) or ApoSOD1 (400 ng/ml/10 min) (g–i). Motor-neuron enriched culture were harvested before the treatment with SOD1 or ApoSOD1. White arrows indicate MAP2-positive neurons with a nuclear localization of p-Akt. Bar graph at the bottom represents the % of p-Akt-positive nuclei in each group. *p < 0.05 versus control. **B** Representative Western blotting and quantification of the effect of SOD1, and ApoSOD1 (both at 400 ng/ml/10 min) on p-Akt and Akt expression in the absence or presence of siNCX1 (10 nmol/L for 48 h). Data are expressed as mean ± SE of three different experimental sessions. *p < 0.05 versus siControl; **p < 0.05 versus siControl, SOD1 and ApoSOD1. **C** Bar graph depicting the effect of L-BMAA (300 μM/48 h) on cell survival in rat primary motor neurons pretreated with SOD1, or ApoSOD1 (both at 400 ng/ml/10 min) in the presence or absence of siNCX1 or after exposure to the NCX1 activator CN-PYB2 (10 nM). Data are expressed as mean ± S.E. of three different experimental sessions. *p < 0.05 versus control; **p < 0.05 versus L-BMAA alone; ***p < 0.05 versus L-BMAA + SOD1 or L-BMAA + ApoSOD1

To clarify the role of NCX1 activation in SOD1 and ApoSOD1 neuroprotective mechanism, Akt localization and expression were studied in primary motor neurons exposed to these molecules. Figure 5A shows a peculiar nuclear localization of active Akt (phospho-Akt; p-Akt) in a subset of MAP2-positive neurons exposed to SOD1 or ApoSOD1 for 10 min. Of note, this subset of motor neurons resembled those small group of cells expressing NCX1 at higher level (please see arrows in Additional File 1). Interestingly, Western blotting analysis showed that SOD1- and ApoSOD1-induced p-Akt overexpression was prevented in primary motor neurons silenced for NCX1 (Fig. 5B). In accordance with these results, siNCX1 prevented both SOD1 and ApoSOD1-induced neuroprotection in primary motor neurons exposed to the neurotoxin L-BMAA (300 µM/48 h) (Fig. 5C; Additional File 3). Of interest, L-BMAA may share the same detrimental mechanism with other cycad toxins inducing downstream mitochondrial dysfunction and reactive oxygen species (ROS) production [35, 36]. Therefore, the effect of SOD1 was tested also in motor neurons exposed to chemical hypoxia, a stimulus for ROS generation (Additional File 2). In this model, SOD1 prevented cell death in a concentration-dependent way by stimulating MEK1/PI3′K/Akt phosphorylation (Additional File 2). As previously demonstrated [12], this protective pathway is activated also by SOD1 in motor neurons exposed to L-BMAA able to induce cell death in a concentration-dependent way (Additional File 3).

Indeed, the participation of NCX1 was further confirmed by the neuroprotective effect of the specific activator of the exchanger isoform CN-PYB2 [24] that prevented L-BMAA-induced cell death in motor neuron-enriched cultures (Fig. 5C).

Collectively, our results demonstrated the important role played by NCX1 in triggering SOD1- and ApoSOD1-dependent prosurvival pathway through an increase of [Ca^2+^]_i_.

## Discussion

With the aim to identify new druggable targets in ALS, the present study provides a comprehensive analysis of the upstream mechanisms underlying SOD1-induced neuroprotection in an in vitro model of the disease. Here, we tested the involvement of P_2_X_7_, NCX, and cADPR, three ionic proteins mainly involved in neuronal [Ca^2+^]_i_ handling and possibly mediating the toxic effect of L-BMAA. For instance, the lack of P_2_X_7_ aggravates ALS symptoms by determining gliosis and motor neuron death [37–41]. Furthermore, NCX dysfunction intervenes in ALS pathogenesis while its activation may prolong life span of SOD1^G93A^ mice through the attenuation of motor neuron loss [31, 42]. On the other hand, cADPR causes Ca^2+^ mobilization [43] through a direct or indirect release from ER [44]. Moreover, in L-BMAA-treated cultures SOD1 produced neuroprotective effects in a Ca^2+^-related way and independently from its catalytic activity [12]. Accordingly, its free-metal form ApoSOD1 may mimic SOD1 effect in L-BMAA-treated cultures by promoting a Ca^2+^-dependent activation of ERK1/2 and Akt and preventing ER stress-induced cell death [12]. Among the ionic mechanisms investigated, we identified the bi-directional ion transporter NCX1 as the unique protein underlying SOD1- and ApoSOD1-induced [Ca^2+^]_i_ increase and, therefore, involved in their prosurvival effects. Patch-clamp experiments revealed that SOD1 as well ApoSOD1 promoted a rapid activation of NCX1 in the reverse mode of operation thus eliciting a significant increase in ER Ca^2+^ content. This possibly counteracted ER Ca^2+^ leak induced by L-BMAA thus delaying ER stress. Of particular interest is that NCX plays a crucial role against ER stress in other neurodegenerative disease including stroke and Alzheimer’s disease [16, 19, 29, 30]. This seems to be due to the ability of the exchanger to counteract Ca^2+^ leak of the most relevant Ca^2+^-storing organelle and, therefore, to hamper the transductional cascade of ER stress. In fact, in an in vitro model of stroke, augmented ER Ca^2+^ refilling was mediated by NCX1 working in the reverse mode [29]. The same may occur in cortical neurons exposed to ischemic preconditioning able to induce tolerance against a subsequent harmful stimulus [30]. This suggests that the antiporter is crucial for counterbalance the ER Ca^2+^ dysfunction induced by hypoxia in neurons. In accordance with this view, in the present study, NCX1 knocking down in primary motor neurons not only reduced ER Ca^2+^ content above the resting level but also induced the overexpression of GRP78, an indubitable ER stress marker. Moreover, the relevance of NCX1 at motor neuron level was confirmed by the neuroprotective effect exerted by the new selective pharmacological activator of NCX1, CN-PYB2 [24], in L-BMAA-treated motor neurons.

Besides its role in mediating the upstream Ca^2+^ increase, NCX expression is regulated by most of the transductional elements activated by SOD1 and ApoSOD1 in motor neurons [12, 45, 46]. On the other hand, by a feedback mechanism, the same transductional elements are modulated by NCX function [12, 47]. This is consistent with the possible long-lasting participation of NCX1 in the transductional cascade underlying the neuroprotective effects of SOD1. In this context, our data showed a peculiar nuclear localization of active Akt in a subset of MAP2-positive neurons exposed to SOD1 as well as ApoSOD1. Interestingly, all Akt forms (i.e. Akt1/2/3) have been reported to reside in the nucleus or to migrate into the nucleus in response to a variety of protective stimuli in order to block apoptotic machinery or to induce the expression of those genes involved in cell survival [48].

Furthermore, we showed that in SBFI-loaded motor neurons, SOD1 as well as ApoSOD1 induced a significant increase in [Na^+^]_i_. In this respect, we reasoned that this ionic mechanism could be useful to drive SOD1-induced activation of NCX1 in the reverse mode of operation. Therefore, it is possible that SOD1 and ApoSOD1 interfered with the Na^+^-dependent NCX1 function by the modulation of other sodium transporters expressed in motor neuron plasma membrane. In this respect, reduced Na^+^/K^+^ ATPase-α3 activity has been observed in animal models of ALS as well as its reduced levels in the spinal cord of both sporadic and familial ALS patients [49]. In addition, the pharmacological inhibition of Na^+^/K^+^ ATPase-α3 is able to worsen disease pathology, thus confirming that an early Na^+^-dependent hyperexcitability is neuroprotective in ALS [50].

Collectively, this study shows that the initial phase of the complex mechanism shared by SOD1 and its non-metalled form ApoSOD1 in ALS/PDC model passed through the activation of NCX1 reverse mode/ER Ca^2+^ refilling and nuclear Akt activation.

## Conclusions

In the present study the Na^+^/Ca^2+^ exchanger isoform 1 (NCX1) has been identified as the main upstream mechanism underlying the non-enzymatic and neuroprotective action of SOD1 in an in vitro model of ALS. Molecularly, P_2_X_7_ receptor and cADP-ribose receptor are not involved in this neuroprotective mechanism. Under basal conditions, a significant co-localization between NCX1 and endogenous SOD1 was observed at plasma membrane level in a motor neuron-enriched culture. Transductionally, SOD1 and ApoSOD1 elicited the activation of NCX1 in the reverse mode of operation favoring Ca^2+^ influx via a previous increase in [Na^+^]_i_. Then, NCX1 recharged ER of Ca^2+^ protecting from ER stress and determining Akt phosphorylation and its nuclear translocation in a subset of primary motor neurons. Furthermore, pharmacological activation of NCX1 protected motor neurons from the toxic effect of L-BMAA thus showing a good profile as a new candidate for pioneering ALS treatment.

## Supplementary Information

**Additional file 1.** (**A**) Immunolocalization of NCX1 (a,d) and MAP2 (b,e) within a motor-neuron enriched culture under control conditions. Nuclei were stained with nuclear DNA stain 4, 6-diamino-2-phenylinndole (DAPI). Arrows indicate MAP2-positive cells with higher level of NCX1 expression. (**B**) Immunolocalization of NCX1 and NCX3 in differentiated NSC-34 cells. (**C**) Quantification of SOD1-induced [Ca^2+^]i in presence of CNQX (20 μM), MK801 (10 μM), or CB-DMB (1 μM) in motor neurons expressed as △% of increase. All the experiments were repeated at least three times on at least 35 cells for each group; *p < 0.001 vs control (basal values of [Ca^2+^]i) .

**Additional file 2.** (**A**) Bar graph depicting the effect of Chemical Hypoxia on cell survival of differentiated NSC-34 cells pretreated (10 min) with 40, 400 or 4000 ng/ml SOD1. Data are expressed as mean±S.E. of three different experimental sessions. *p<0.05 versus control; **p<0.05 versus Chemical Hypoxia alone and ***p<0.05 versus All. (**B**) DCF-DA-detected ROS production in differentiated NSC-34 cells exposed to Chemical Hypoxia or Chemical Hypoxia plus SOD1 (400 ng/ml). Data are expressed as mean±S.E. of three different experiments. *p<0.05 versus control; **p<0.05 versus Chemical Hypoxia alone. (**C**) Bar graph depicting the effect of Chemical Hypoxia on cell survival of differentiated NSC-34 motor neurons transfected with siMEK1 (10 nM) or treated with PD98059, or Akt D− (2 μg/μl) or treated with LY294002 and then exposed to SOD1 (400 ng/ml/10 min). Data are expressed as mean±S.E. of three different experimental sessions. *p<0.05 versus control alone; **p<0.05 versus Chemical Hypoxia alone; ***p<0.05 versus Chemical Hypoxia +SOD1.

**Additional file 3.** Bar graph depicting the effect of L-BMAA (0.01-1 mM) on cell survival of differentiated NSC-34 cells. Data are expressed as mean±S.E. of three different experimental sessions. *p<0.05 versus control or 0.01 mM and 0.1 mM L-BMAA; **p<0.05 versus control and all previous concentrations.

## Data Availability

All raw data are available on request.

## Abbreviations

[Ca^2^^+^]_Ii_: Intracellular calcium concentration
ER: Endoplasmic reticulum
SOD1: Cu,Zn-superoxide dismutase
NCX1: Na^+^/Ca^2^^+^ exchanger isoform 1
NCX3: Na^+^/Ca^2^^+^ exchanger isoform 3
I_NCX_: NCX currents
L-BMAA: Hydrochloride/β-*N*-methylamino-l-alanine
ALS: Amyotrophic lateral sclerosis
MTT: 3-(4,5-Dimethylthiazol-2-yl)-2,5, diphenyltetrazolium bromide
Fura-2: (1-[2-(5-Carboxyoxal-2-yl)-6-aminobenzofuran-5-oxyl]-2-(2′-amino-5′-methylphenoxy)-ethane-*N*,*N*,*N*′,*N*′-tetraacetic acid)
SBFI-AM: 1,3-Benzenedicarboxylic acid, 4,4′-[1,4,10-trioxa-7,13-diazacyclopentadecane-7,13-diylbis(5-methoxy-6,12-benzofurandiyl)]bis-,tetrakis[(acetyloxy)methyl] ester
