# Supplementary material for: Na+/Ca2+ exchanger isoform 1 takes part to the Ca2+-related prosurvival pathway of SOD1 in primary motor neurons exposed to beta-methylamino-l-alanine
Source: Cell Commun Signal. 2022 Jan 12;20:8. doi: 10.1186/s12964-021-00813-z (PMC8756626; doi:10.1186/s12964-021-00813-z)
Supplement: Supplementary file 2 — Additional file 1. (A) Immunolocalization of NCX1 (a,d) and MAP2 (b,e) within a motor-neuron enriched culture under control conditions. Nuclei were stained with nuclear DNA stain 4, 6-diamino-2-phenylinndole (DAPI). Arrows indicate MAP2-positive cells with higher level of NCX1 expression. (B) Immunolocalization of NCX1 and NCX3 in differentiated NSC-34 cells. (C) Quantification of SOD1-induced [Ca2+]i in presence of CNQX (20 μM), MK801 (10 μM), or CB-DMB (1 μM) in motor neurons expressed as △% of increase. All the experiments were repeated at least three times on at least 35 cells for each group; *p < 0.001 vs control (basal values of [Ca2+]i) . [file 12964_2021_813_MOESM2_ESM.pdf]

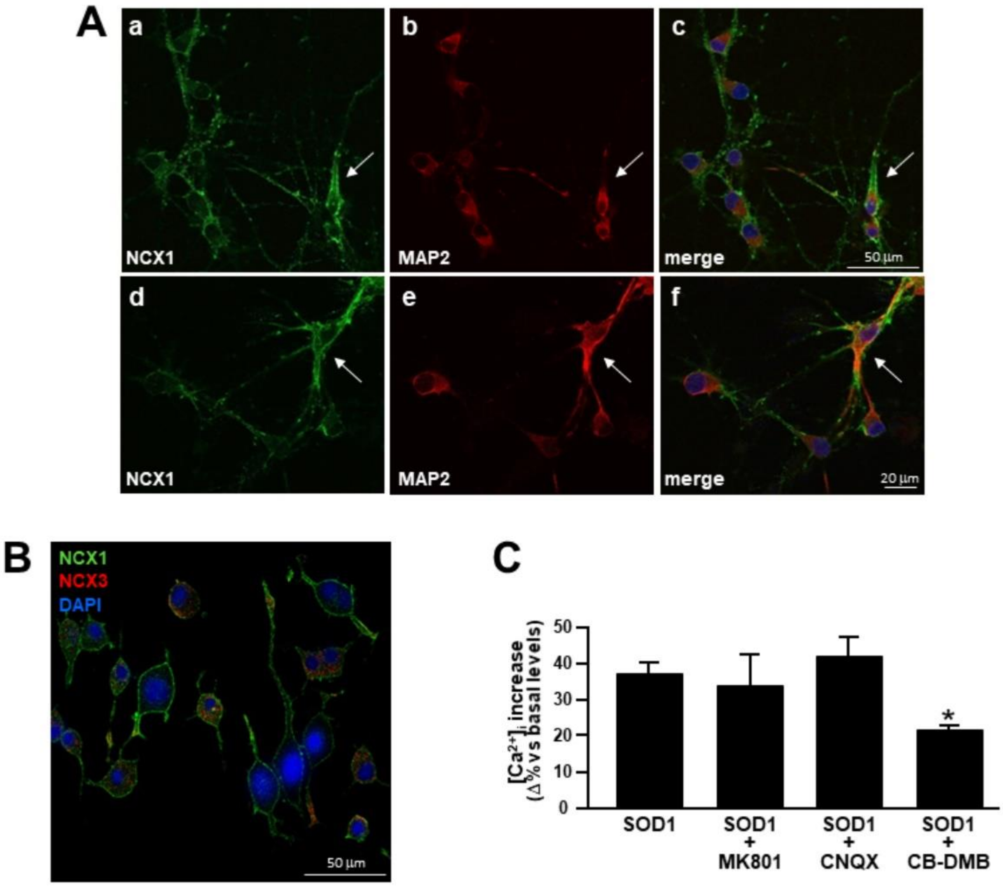

Additional File 1. (**A**) Immunolocalization of NCX1 (a,d) and MAP2 (b,e) within a motor-neuron enriched culture under control conditions. Nuclei were stained with nuclear DNA stain 4, 6-diamino-2-phenylinndole (DAPI). Arrows indicate MAP2-positive cells with higher level of NCX1 expression. (**B**) Immunolocalization of NCX1 and NCX3 in differentiated NSC-34 cells. (**C**) Quantification of SOD1-induced $[Ca^{2+}]_i$ in presence of CNQX (20 µM), MK801 (10 µM), or CB-DMB (1 µM) in motor neurons expressed as Δ% of increase. All the experiments were repeated at least three times on at least 35 cells for each group; *$p < 0.001$ vs control (basal values of $[Ca^{2+}]_i$) .
