# Supplementary material for: Na+/Ca2+ exchanger isoform 1 takes part to the Ca2+-related prosurvival pathway of SOD1 in primary motor neurons exposed to beta-methylamino-l-alanine
Source: Cell Commun Signal. 2022 Jan 12;20:8. doi: 10.1186/s12964-021-00813-z (PMC8756626; doi:10.1186/s12964-021-00813-z)
Supplement: Supplementary file 3 — Additional file 2. (A) Bar graph depicting the effect of Chemical Hypoxia on cell survival of differentiated NSC-34 cells pretreated (10 min) with 40, 400 or 4000 ng/ml SOD1. Data are expressed as mean±S.E. of three different experimental sessions. *p<0.05 versus control; **p<0.05 versus Chemical Hypoxia alone and ***p<0.05 versus All. (B) DCF-DA-detected ROS production in differentiated NSC-34 cells exposed to Chemical Hypoxia or Chemical Hypoxia plus SOD1 (400 ng/ml). Data are expressed as mean±S.E. of three different experiments. *p<0.05 versus control; **p<0.05 versus Chemical Hypoxia alone. (C) Bar graph depicting the effect of Chemical Hypoxia on cell survival of differentiated NSC-34 motor neurons transfected with siMEK1 (10 nM) or treated with PD98059, or Akt D− (2 μg/μl) or treated with LY294002 and then exposed to SOD1 (400 ng/ml/10 min). Data are expressed as mean±S.E. of three different experimental sessions. *p<0.05 versus control alone; **p<0.05 versus Chemical Hypoxia alone; ***p<0.05 versus Chemical Hypoxia +SOD1. [file 12964_2021_813_MOESM3_ESM.pdf]

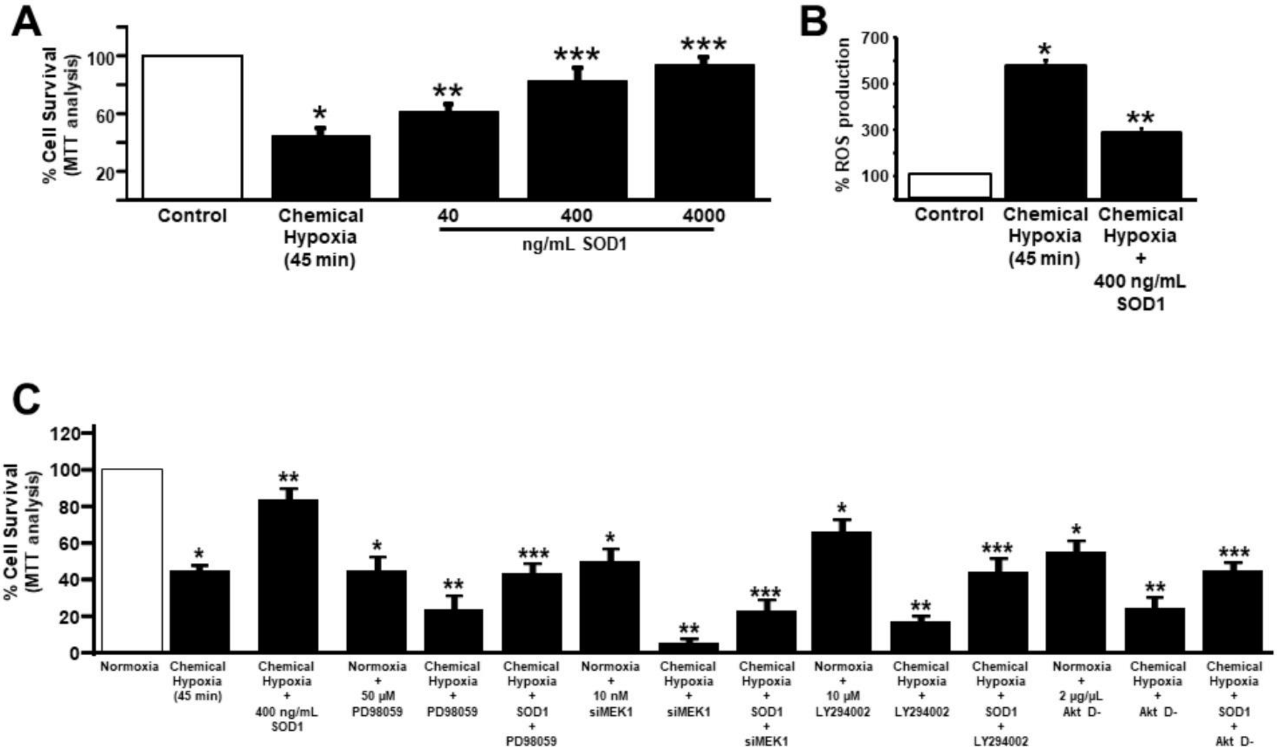

Additional File 2. **(A)** Bar graph depicting the effect of Chemical Hypoxia on cell survival of differentiated NSC-34 cells pretreated (10 min) with 40, 400 or 4000 ng/ml SOD1. Data are expressed as mean±S.E. of three different experimental sessions. *p<0.05 versus control; **p<0.05 versus Chemical Hypoxia alone and ***p<0.05 versus All. **(B)** DCF-DA-detected ROS production in differentiated NSC-34 cells exposed to Chemical Hypoxia or Chemical Hypoxia plus SOD1 (400 ng/ml). Data are expressed as mean±S.E. of three different experiments. *p<0.05 versus control; **p<0.05 versus Chemical Hypoxia alone. (**C**) Bar graph depicting the effect of Chemical Hypoxia on cell survival of differentiated NSC-34 motor neurons transfected with siMEK1 (10 nM) or treated with PD98059, or Akt D− (2 µg/µl) or treated with LY294002 and then exposed to SOD1 (400 ng/ml/10 min). Data are expressed as mean±S.E. of three different experimental sessions. *p<0.05 versus control alone; **p<0.05 versus Chemical Hypoxia alone; ***p<0.05 versus Chemical Hypoxia +SOD1.
