# Supplementary material for: Na+/Ca2+ exchanger isoform 1 takes part to the Ca2+-related prosurvival pathway of SOD1 in primary motor neurons exposed to beta-methylamino-l-alanine
Source: Cell Commun Signal. 2022 Jan 12;20:8. doi: 10.1186/s12964-021-00813-z (PMC8756626; doi:10.1186/s12964-021-00813-z)
Supplement: Supplementary file 4 — Additional file 3. Bar graph depicting the effect of L-BMAA (0.01-1 mM) on cell survival of differentiated NSC-34 cells. Data are expressed as mean±S.E. of three different experimental sessions. *p<0.05 versus control or 0.01 mM and 0.1 mM L-BMAA; **p<0.05 versus control and all previous concentrations. [file 12964_2021_813_MOESM4_ESM.pdf]

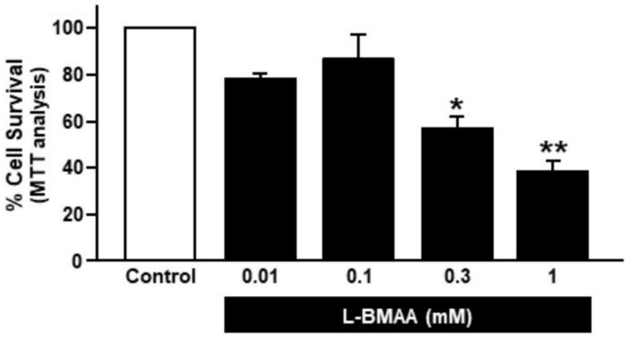

Additional File 3. Bar graph depicting the effect of L-BMAA (0.01-1 mM) on cell survival of differentiated NSC-34 cells. Data are expressed as mean±S.E. of three different experimental sessions. *$p<0.05$ versus control or 0.01 mM and 0.1 mM L-BMAA; ** $p<0.05$ versus control and all previous concentrations.
